# Mucinous adenocarcinoma with peritoneal carcinomatosis presenting as acute abdomen: a case report

**DOI:** 10.3389/fonc.2025.1722960

**Published:** 2026-02-04

**Authors:** Resul Nusretoğlu, Abdullahi Khalif Ali

**Affiliations:** 1 General Surgery Department, Mogadishu Somali–Türkiye Recep Tayyip Erdoğan Training and Research Hospital, Mogadishu, Somalia; 2 Benadir University, Mogadishu, Somalia

**Keywords:** acute abdomen, mucinous cystadenocarcinoma, ovarian cancer, peritoneal carcinomatosis, salpingo-oophorectomy

## Abstract

**Background:**

Ovarian mucinous cystadenocarcinoma is a rare malignant epithelial tumor, accounting for approximately 10–15% of ovarian carcinomas. It typically presents with nonspecific abdominal symptoms and may reach a large size before detection. Presentation as an acute abdomen is uncommon and usually reflects advanced disease complicated by rupture or extensive peritoneal carcinomatosis.

**Case Presentation:**

A 70-year-old postmenopausal woman presented with severe abdominal pain, vomiting, and fever. Laboratory evaluation revealed marked leukocytosis and elevated inflammatory markers. Imaging demonstrated a large multiloculated left adnexal cystic mass measuring 20 × 12 cm, associated with massive ascites and omental thickening. Due to signs of acute abdomen and clinical deterioration, urgent exploratory laparotomy was performed. Intraoperatively, diffuse peritoneal carcinomatosis and a large left ovarian mass were identified, and approximately 12 liters of mucinous fluid were drained. The patient underwent total abdominal hysterectomy with bilateral salpingo-oophorectomy, infracolic omentectomy, and appendectomy. Histopathological examination confirmed mucinous adenocarcinoma. The postoperative course was uneventful, and the patient demonstrated good recovery at six-month follow-up.

**Conclusion:**

Ovarian mucinous adenocarcinoma may rarely present as an acute abdomen due to extensive peritoneal involvement. Early recognition, thorough exclusion of alternative acute abdominal etiologies, accurate histopathological diagnosis, and multidisciplinary management are essential for optimal outcomes.

## Introduction

Mucinous tumors constitute approximately 10–15% of epithelial ovarian neoplasms, with only 3–5% being malignant mucinous cystadenocarcinomas (1–3). These tumors arise from the ovarian surface epithelium and are characterized by large multilocular cystic lesions lined with mucin-producing malignant epithelial cells demonstrating stromal invasion (4, 5). Ovarian mucinous adenocarcinoma is a rare epithelial malignancy that represents a biologically distinct disease entity with unique clinicopathological characteristics, typically presenting with nonspecific symptoms and often reaching a large size before diagnosis (5). They are typically unilateral and often exceed 20 cm in diameter at presentation (6).

Clinical symptoms are frequently nonspecific, including abdominal distension, pelvic pain, or a palpable mass, leading to delayed diagnosis (7). Imaging modalities such as ultrasonography and computed tomography (CT) can detect complex ovarian masses but cannot reliably distinguish benign, borderline, and malignant lesions (8). Consequently, histopathological examination remains the gold standard for diagnosis (9).

Acute abdomen is a rare presentation and generally indicates advanced disease complicated by rupture, hemorrhage, or peritoneal carcinomatosis. We report a case of primary ovarian mucinous adenocarcinoma presenting as acute abdomen, highlighting diagnostic challenges and management considerations.

## Case presentation

### Patient history

A 70-year-old postmenopausal woman presented to the emergency department with severe abdominal pain, vomiting, fever, and rebound tenderness. Her past medical history was unremarkable, with no chronic illnesses, prior abdominal or pelvic surgeries, or gynecologic disorders. There was no personal or family history of ovarian, breast, or colorectal malignancy. She was not taking any regular medications and had no known drug allergies. She denied smoking, alcohol consumption, or illicit drug use. Gynecological history revealed gravida 8, para 8 (G8P8), with no history of hormone replacement therapy or infertility treatment.

### Clinical findings

On admission, vital signs showed a temperature of 38.7 °C, heart rate of 150 beats/min, blood pressure of 108/69 mmHg, respiratory rate of 22 breaths/min, and oxygen saturation of 95% on room air. Physical examination demonstrated marked abdominal distension extending to the xiphoid process, with a large palpable intra-abdominal mass. Diffuse tenderness, guarding, and rebound tenderness were present in all quadrants.

Laboratory investigations revealed leukocytosis (WBC 19,000/µL) and markedly elevated C-reactive protein (200 mg/L). Blood and urine cultures were negative.

The fever and leukocytosis were interpreted as manifestations of an acute inflammatory response related to tumor rupture and extensive peritoneal carcinomatosis rather than a primary infectious process. This was supported by the absence of a focal infectious source on imaging and intraoperative assessment, as well as rapid postoperative resolution without prolonged antibiotic therapy.

### Imaging and differential diagnosis

**Figure 1:**
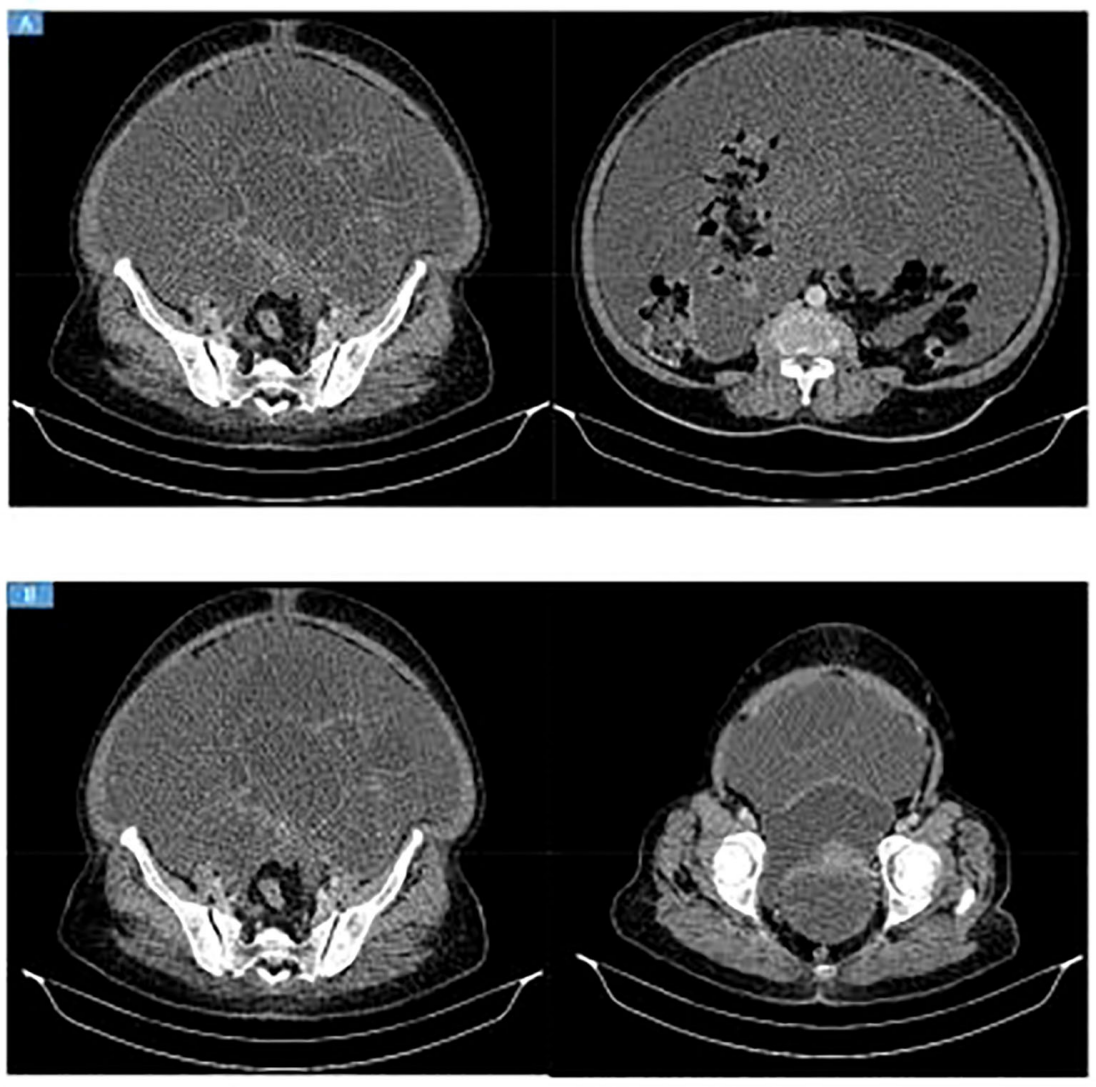
**(A, B)** Contrast-enhanced CT showing massive intra-abdominal fluid collectionwith thick, septated, enhancing areas predominantly in the lower abdomen and pelvis, associated with omental thickening and mild fat stranding. A large left adnexal lesionmeasuring approximately 20 × 12 cm is seen, containing enhancing solid components andinternal septations, causing posterior compression of the rectum. Findings are highly suggestive of ovarian malignancy, most likely mucinous cystadenocarcinoma.

Abdominal and pelvic ultrasonography revealed a large multiloculated, thick-walled cystic mass of probable adnexal origin. Contrast-enhanced CT demonstrated a left adnexal mass measuring approximately 20 × 12 cm with enhancing solid components and internal septations, accompanied by massive ascites, omental thickening, mild fat stranding, and posterior rectal compression (**Figure 1**).

Prior to surgery, a broad differential diagnosis for acute abdomen was considered. Gastrointestinal causes such as bowel perforation, intestinal obstruction, ischemia, and complicated appendicitis were evaluated and excluded radiologically. Gynecologic causes including adnexal torsion, ruptured ovarian cyst, and tubo-ovarian abscess were also considered. Adnexal torsion was ruled out based on preserved vascular enhancement on CT and absence of a twisted pedicle. Postoperative or surgical-site–related complications were excluded due to the absence of prior abdominal surgery.

### Surgical findings

**Figure 2:**
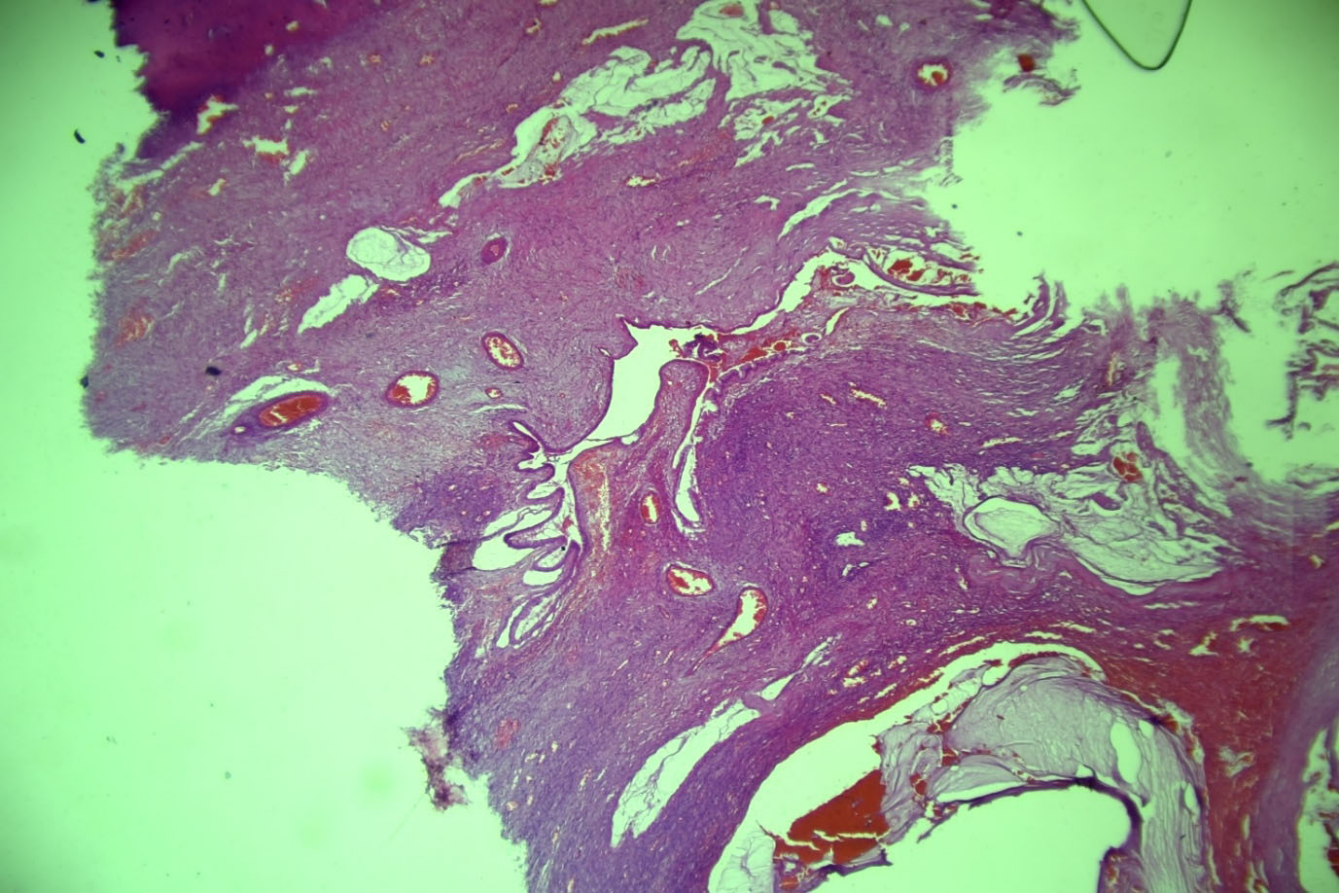
The 4× H&E stained section demonstrates extracellular mucin with mucinous infiltration involving the endocervical tissue.

Due to clinical deterioration and signs of acute abdomen, urgent exploratory laparotomy was performed. Intraoperatively, approximately 12 liters of mucinous fluid were aspirated and sent for cytological analysis. No abscess formation, purulent collections, or signs of bacterial peritonitis were identified. Diffuse peritoneal carcinomatosis with metastatic implants on the peritoneum and diaphragm was observed, along with a large left ovarian mass measuring approximately 20 cm. The uterus, right adnexa, and appendix appeared grossly normal (**Figure 2**).

Pseudomyxoma peritonei was considered intraoperatively; however, the absence of characteristic gelatinous mucin deposits coating peritoneal surfaces and the lack of diffuse mucinous implants argued against this diagnosis.

### Surgical management

The patient underwent peritoneal fluid sampling, total abdominal hysterectomy with bilateral salpingo-oophorectomy, infracolic omentectomy, and appendectomy. Cytoreductive surgery was deliberately limited following intraoperative assessment. Although disease was advanced, achieving complete macroscopic cytoreduction would have required extensive multiorgan resection. Given the patient’s advanced age and elevated perioperative risk, the surgical strategy prioritized symptom relief and reduction of morbidity rather than aggressive maximal cytoreduction.

### Postoperative course and oncologic management

**Figure 3:**
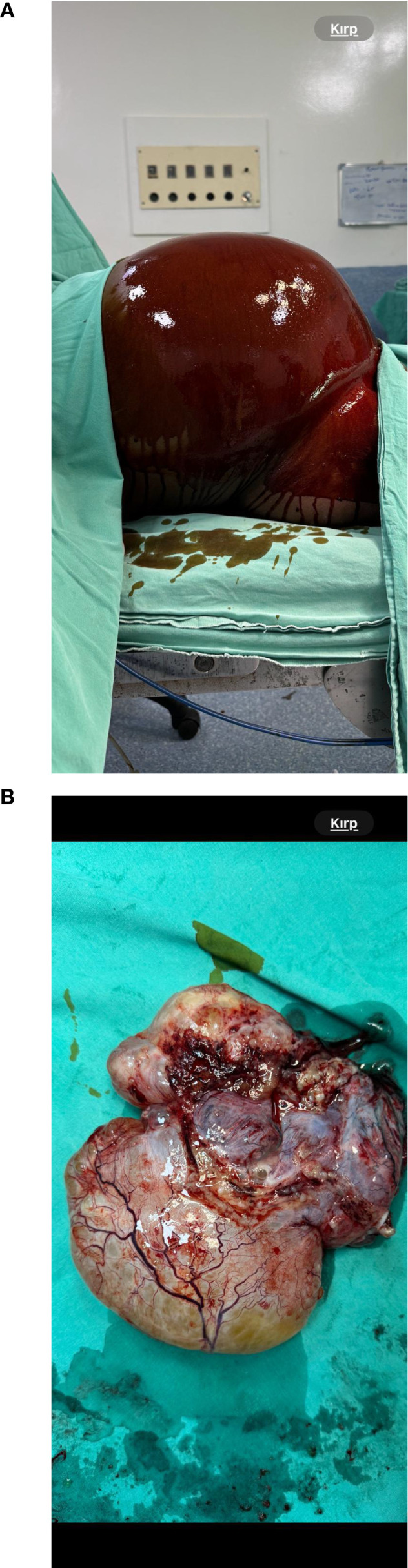
**(A)** (4×, H&E): Section showing extracellular mucin with mucinous infiltrationinvolving endocervical tissue. **(B)** (10×, H&E): Higher magnification demonstrating extracellular mucin andmucinous infiltration within endocervical tissue.

The patient was transferred to the intensive care unit postoperatively in stable condition. Her recovery was uneventful, with return of bowel function on postoperative day six. She was discharged on postoperative day ten. Follow-up visits at one, two, and six months demonstrated continued clinical improvement without complications (**Figure 3**).

Cytological examination of the peritoneal fluid was positive for malignant cells consistent with mucinous adenocarcinoma. Histopathological analysis confirmed extracellular mucin with malignant mucinous epithelial infiltration. There was no histological evidence supporting pseudomyxoma peritonei, and appendiceal pathology excluded a gastrointestinal primary tumor.

Following multidisciplinary oncology consultation, adjuvant chemotherapy was discussed. Considering the advanced stage of disease, histopathological findings, and the patient’s functional status, adjuvant platinum-based chemotherapy was administered in accordance with established oncologic protocols. The patient tolerated treatment well, with no significant treatment-related adverse effects during early follow-up.

## Discussion

Ovarian mucinous adenocarcinoma is a rare epithelial malignancy that typically presents with nonspecific symptoms and may reach a large size before diagnosis. Presentation as an acute abdomen is uncommon and usually reflects advanced disease with rupture or extensive peritoneal carcinomatosis.

In this case, fever and leukocytosis initially raised concern for intra-abdominal infection. However, comprehensive laboratory testing, imaging studies, and intraoperative findings excluded infection, supporting a tumor-related inflammatory etiology. Careful preoperative evaluation was essential to exclude other causes of acute abdomen, including gastrointestinal perforation, adnexal torsion, and postoperative complications.

Differentiation from pseudomyxoma peritonei is critical in mucinous tumors with ascites. In our patient, the absence of characteristic gelatinous peritoneal deposits and histopathological confirmation of malignant mucinous epithelial cells supported the diagnosis of primary ovarian mucinous adenocarcinoma with peritoneal carcinomatosis.

While maximal cytoreduction is associated with improved survival, individualized surgical decision-making is necessary in elderly patients. Limited cytoreduction was appropriate in this case to minimize morbidity while achieving symptom control. Adjuvant chemotherapy remains the standard of care for advanced-stage disease, although mucinous tumors are known to exhibit relative chemoresistance.

### Limitations

Several limitations of this case report should be acknowledged. Management decisions were influenced by resource constraints, which may have limited access to advanced diagnostic and therapeutic options. As a single case, findings cannot be generalized. Additionally, follow-up was limited to six months, preventing assessment of long-term outcomes such as recurrence, progression-free survival, and overall survival. Patient-reported quality-of-life outcomes were not systematically documented.

## Conclusion

Ovarian mucinous adenocarcinoma is a rare malignancy that can present as an acute abdomen due to extensive peritoneal involvement. Early recognition, exclusion of alternative acute abdominal etiologies, accurate histopathological diagnosis, and multidisciplinary management are essential. Individualized surgical strategies and appropriate oncologic therapy are particularly important in elderly patients with advanced disease.

## Data Availability

The datasets presented in this study can be found in online repositories. The names of the repository/repositories and accession number(s) can be found below: no.
